# Supplementary material for: The Burden of Treatment: Experiences of Patients Who Have Undergone Radiotherapy and Proton Beam Therapy
Source: Healthcare (Basel). 2025 Jun 5;13(11):1351. doi: 10.3390/healthcare13111351 (PMC12155157; doi:10.3390/healthcare13111351)
Supplement: Supplementary file 1 [file healthcare-13-01351-s001.zip › Table S1 Additional data.pdf]

Table S1. Supporting quotes for themes and subthemes

Theme	Subtheme	Supporting quotes
Informational needs	Information and guidance during treatment	➢ 'I was able to access all the information, particularly those Macmillan books, they are helpful, very, very clear.' - PR16<br>➢ 'He [the consultant] went through all the side effects and when I came out of that, I remember thinking Oh my God I just don't know if I wanted to hear all of that, but they have to say it all of course. Sometimes you hear so much that it worries you more than anything else.' - PR01<br>➢ 'They try to explain everything because you are new to your department, right? So they try to explain everything because before they do every step. So, it makes you feel that more relief, at least you know what's happening.' - PR-02
	Information and guidance post-treatment	➢ 'You end up doing it on your own, without any guidance.' - PR-27<br>➢ 'I'm at that point now where I'm like, has it gone away, or is that still chipping away at me inside? But that does bother me. Some days. I'm like, what's happening now?' - PR20<br>➢ 'Yeah, I had it every week (Physio/OT). And that was great. That was great. But it's interesting, because actually, when I need it, most is post-treatment... So yeah, it potentially might have been helpful to see them again.' - PR08<br>➢ 'Obviously, I was leaving my little team supporting me for six weeks.' - PR03
Holistic needs	Impact on mood	➢ 'But I think the mental aspect of it was draining and hard. I think I got more frustrated as it went on. Week Four it was tough, the whole two weeks I wasn't in a happy place I've really struggled with is the mental aspect.' - PR03<br>➢ 'I have noticed that the mood is up and then down a little bit, even more [afterwards] than when I was going through treatment actually somehow, yeah.' - PR01
	Increased anxiety	➢ 'I was anxious. But I think it was just because I didn't know what to expect when I was in the room. I remember being really anxious and nearly teary...I think it's probably the realization of what's happening.' - PR01<br>➢ 'And then my skin started to peel quite a lot. I started going into a panic stage. I was like, oh my god, I'm feeling a lot like now.' - PR15<br>➢ 'So I'm not someone who's claustrophobic at all. Yeah, I cannot say that that thought has ever crossed my mind. But I must say having a mask fitted and then being put through the MRI scanner. I had a little like a slight moment of panic.' - PR08<br>➢ 'Because like I said, there were some dark moments. And I suppose the biggest one is I don't know what lies ahead.' - PR-18
	Hope and Optimism	➢ 'I appreciate how lucky and I have been right, so there's no denying that in terms of I been an impact the physical symptoms I had made it easy to pick up what the issue was, got scanned quickly, got referred quickly to [city name], had an amazing world expert deal with it, lucky enough to have like superb proton beam therapy which very few people get all that say.' - PR26<br>➢ 'I've been quite positive, positive about it. We've almost got that attitude of, you know, it is what it is and what will happen will happen. But sort of staying positive with its treatment at the moment, it's still potentially curative intent. Yes. Stay positive with everything, and you sort of trust the experts.' - PR23
	Importance of faith and spirituality	➢ So the whole experience has helped to reconnect with that [faith]. That's what definitely gave me the positivity which everyone's said I've had. If it wasn't for that faith, that strength to try to always see the bigger picture.' - PR26<br>➢ 'Every time I was lying down on the bed while it was happening close my eyes I was praying.' - PR15<br>➢ 'I asked them for a longer gown. Because I didn't like you know, I mean in my religion, if you just ask them, they will do it. They will make sure you are comfortable.' - PR15
	Change in appearance	➢ 'It was the loss of beard that was the challenge. I've had a beard since 1980s and I won't be able to have a beard anymore.' - PR05<br>➢ 'Yeah, well, if you're losing weight, you get to a point where you're not happy about how much you lost? Yeah, so I think the moral of the story is to be just happy with what you've got' - PR03<br>➢ 'So yeah, I didn't have a great sort of self-esteem, about the way I looked. Also the weight gain. So a lot of my clothes didn't fit me.' - PR14

Physical impacts	Symptoms worsening over time	➢ 'I think the skin start to get worse at the last week of the treatment. Yeah, last week of the treatment. And then after the treatment as well. Yeah. Yeah. Like, I went to the two to three weeks after the treatment. That is when it was the most painful.' - PR02<br>➢ 'From two to three I start to feel I'm getting sore and by then by the time I got to week three I stopped eating. Week four my mouth was just horrendous I couldn't eat anything because just I just wanted to be sick I couldn't swallow.' – PR03<br>➢ 'It actually hit me about roughly about a week afterwards. And then there was a period of about one week where I was in bed most of the time.' - PR07
	Difficulty managing symptoms	➢ 'The fatigue. That was the worst. And then I could barely stand. I could barely stand or go for short walks, I was so exhausted.' - PR08<br>➢ 'I had sort of a bit of pain in the sort of oesophagus and sort of acid. Generally on the night time when you're sort of lying down. Yeah, this is what wakes you up just as you're drifting off.' - PR23<br>➢ 'Like five weeks into the treatment I was. I didn't get caught short. I was very close to it at the train station sometimes because it in a heartbeat is like you need to go to the toilet. And it's like, Where the hell's the toilet?' - PR22<br>➢ 'I didn't feel the side effects physically until week 3 1/2 or 4 when the skin was very raw and peeling.' - PR01<br>➢ 'The worst one was the mouth ulcers. Yeah, yeah, that was. That was the worst thing. Uh 'cause that made it difficult to eat. Quite painful actually.' - PR07
	Physical comfort during treatment	➢ 'Oh, it's horrible... It was the fact that I had to like they said I had to lie perfectly still without moving right. And that's fine for a few minutes. But it actually took 40 minutes. Right. I had to lie completely still not move at all for 40 minutes with my head clamped down. And after at the end of it, my whole body had sort of gone numb.' PR-07<br>➢ 'For me, lying down on the table with my arms above my head was, was an uncomfortable feeling. It was, it's a sense of vulnerability... It's difficult to get comfortable. You have to hold it for a while. Worst thing.' - PR14<br>➢ 'Holding your water was sometimes a bit uncomfortable, we'll see if, if the treatment was delayed, for whatever reason, you know, sometimes things come up.' - PR19<br>➢ 'I thought it was a bit scary. But it was just the machine going around me and even on me being on the edge of the bed kind of thing.' - PR15<br>➢ 'I didn't like having tattoos. And I was very upset about that... And I don't think it's very nice to give people tattoos. I think that's emotionally quite disturbing. I think it's cruel.' - PR14
	Sexual functioning	➢ 'I can't gain an erection at the moment. But, you know, if it does become a big problem, then then perhaps I'll ask for some help. But at the moment, it's got bigger fish to fry at the moment.' – PR22<br>➢ 'There's no longer an element of physical sexual involvement. I still crave romance and love women. I can't imagine any more sexual feelings for someone. Just the testosterone is gone.' - PR16<br>➢ 'I was going through menopause anyway. And I know that affects that can affect your sex drive. And I know that chemo can also push that push people through like early menopause. So I think that's impact impacted on my sex drive.' - PR14<br>➢ 'I don't feel like as sexual as before, so I'm hoping that comes back. I'm hoping sort of like, you know, builds up, and maybe you know, that will happen once I start exercising [losing weight] and feeling better about myself.' - PR14
	Reduced capacity for things	➢ 'You can't really plan anything on any given day, because it's for me, obviously, it was travelling into London every day at different times every day. Yeah, absolutely. It was almost having to write off five weeks, but I couldn't really think of a way around that.' - PR23<br>➢ 'And I've not got back to walking. I used to spend a lot of time walking before.' - PR03<br>➢ 'You know, that I would spend a couple of hours in the in the hospital every day. That wasn't really a problem. And I think it would have been difficult if I'd still been working or, you know.' - PR-19
Logistical concerns	Coordination and access of care	➢ 'So initially the NHS refused to fund Proton beam. So they said they were gonna do ordinary radiation, right? But fortunately, my health insurance through work said that they would fund it. So they were going to pay for it.' - PR07<br>➢ 'I didn't know who was in charge, and sometimes I get emails saying, well, it's not us anymore, you're with them now.' - PR16<br>➢ 'The NHS doesn't seem to have one NHS databank. For example, I was having to forward emails from one hospital to another hospital because they weren't kept in the loop, or they weren't aware of each other if you'd like.' - PR20

Table S1. Supporting quotes for themes and subthemes

		➢ 'I felt as if I was coordinating amongst all the professionals because I required in order neurology, endocrinology and kind of radiologists for the proton beam therapy, so that in itself was stressful.' - PR26
	Transport and accommodation	➢ 'The biggest issue that I faced was the railways. My son in law drove up to [city name] wants to pick me up because it was no way I was gonna get home for at least another five hours.' - PR20<br>➢ 'Oh, it did cost me quite a lot of money in Uber. So I was worried about public transport, because I was scared of getting sick.' - PR14<br>➢ 'But obviously, you have to stay up there [London] and we came over the weekend. And obviously, the travel costs. Yeah, we had to fork out which is a lot of money. The policy decision is made across the UK is not to help the patient's travel to Manchester or London.' - PR10<br>➢ 'The first week of radiotherapy I did use mini cabs. I spent about two to 300 quid so I couldn't go on like this. So yeah, I went on hospital transport.' PR-30
	Financial burden	➢ 'We ate out one right across the road from the accommodation. I was like I can't do this every night because it's gonna cost a few bucks.' - PR18<br>➢ 'Oh, it did cost me quite a lot of money in Uber. So I was worried about public transport, because I was scared of getting sick.' - PR14<br>➢ 'Yeah, that's, you know, you shouldn't need to worry about that, the financial drain of travelling back and forth.' - PR10<br>➢ 'They introduce the low income financial system or the scheme that you can apply. But we are not eligible on that. But still everyday I have to travel to the hospital and the transportation cost is not that cheap right.' - PR02<br>➢ 'I don't have the kind of income protection. So I did take a hit on income. Yeah. We haven't had financial difficulties, not really. We took a financial hit, but that's different.' - PR05
Interpersonal impacts	Impact on family and friends	➢ 'But you know it, it is taking a toll on them. They've had to put their head down and get on with it and gone through a lot of stuff themselves as well.' - PR26<br>➢ 'You know, I'm not hiding my sort of true results or anything. It's just I don't want to worry her. I filter things.' - PR23<br>➢ 'My wife tries her best but she gets upset more than what I do. So I have to spend tome trying to calm her down.' - PR27
	Patient peer support	➢ 'I met other people that were going through the same thing with me. But obviously they had different areas. I met this lady who had the same time as me on the same place. Just has a little bit up on her leg. It was just nice to meet them, you know just to talk to them.' - PR15
	Relationship with health care professionals	➢ 'I couldn't give them enough credit from the radiographers to the sort of speech and language. All the therapist, dietician all being amazing. And hey, especially radiotherapists, and it's a great team. They saw you through the process.' - PR03<br>➢ 'They play a very Important part to make the whole experience feel, yeah, so friendly. So actually after like, one week, one week's time, you already don't feel nervous. Yeah and very happy to see the similar face.' - PR02
